# Transfer Learning-Enhanced Prediction of Glass Transition Temperature in Bismaleimide-Based Polyimides

**DOI:** 10.3390/polym17131833

**Published:** 2025-06-30

**Authors:** Ziqi Wang, Yu Liu, Xintong Xu, Jiale Zhang, Zhen Li, Lei Zheng, Peng Kang

**Affiliations:** 1 School of Materials Science and Engineering, Beihang University, Beijing 100191, China; qiwzq_work@163.com (Z.W.); xuxintong95@hotmail.com (X.X.); zhangjl315407@163.com (J.Z.); zli@buaa.edu.cn (Z.L.); zhenglei@buaa.edu.cn (L.Z.); 2 State Key Laboratory of Artificial Intelligence for Material Science, Beihang University, Beijing 100191, China; 3 Tianmushan Laboratory, Yuhang District, Hangzhou 311115, China

**Keywords:** bismaleimide-based polyimides, transfer learning, T_g_ prediction, molecular design, machine learning, glass transition temperature

## Abstract

The glass transition temperature (T_g_) was a pivotal parameter governing the thermal and mechanical properties of bismaleimide-based polyimide (BMI) resins. However, limited experimental data for BMI systems posed significant challenges for predictive modeling. To address this gap, this study introduced a hybrid modeling framework leveraging transfer learning. Specifically, a multilayer perceptron (MLP) deep neural network was pre-trained on a large-scale polymer database and subsequently fine-tuned on a small-sample BMI dataset. Complementing this approach, six interpretable machine learning algorithms—random forest, ridge regression, k-nearest neighbors, Bayesian regression, support vector regression, and extreme gradient boosting—were employed to construct transparent predictive models. SHapley Additive exPlanations (SHAP) analysis was further utilized to quantify the relative contributions of molecular descriptors to T_g_. Results demonstrated that the transfer learning strategy achieved superior predictive accuracy in data-scarce scenarios compared to direct training on the BMI dataset. SHAP analysis identified charge distribution inhomogeneity, molecular topology, and molecular surface area properties as the major influences on T_g_. This integrated framework not only improved the prediction performance but also provided feasible insights into molecular structure design, laying a solid foundation for the rational engineering of high-performance BMI resins.

## 1. Introduction

The thermal stability of polymer substrates in high-temperature environments represents a critical technical bottleneck limiting the advancement of advanced composite materials. The glass transition temperature (T_g_), a key parameter governing the transition from the glassy to the rubbery state, directly influences the morphological stability and mechanical property retention of thermoplastic and thermosetting polymers under extreme conditions [1]. For high-performance thermosetting polymers such as bismaleimide-based polyimide (BMI) resins, T_g_ determines their practical application potential in critical fields such as aerospace thermal protection structures and microelectronic packaging materials [2]. However, optimizing T_g_ in BMI resins remains a complex challenge, as it is highly dependent on the composite and architecture of polymers [3,4,5,6]. Traditional trial-and-error approaches are constrained by lengthy experimental cycles, prohibitive testing costs, and difficulties in elucidating microscopic mechanisms. While computational methods like density functional theory (DFT) [7] and molecular dynamics (MD) simulations offer theoretical frameworks for T_g_ prediction [8,9,10], the intricate cross-linking networks, multidimensional aromatization reactions, and synergistic functional group interactions in BMI resins introduce dual challenges, namely exponentially increasing computational resource demands and empirical force field parameter selection in atomic-scale simulations.

Recent advances in polymer informatics have introduced a data-driven paradigm for materials development, enabling an inverse analysis of structure–property relationships through machine learning [11,12,13,14,15]. Notable progress includes Lei et al.’s [16] systematic benchmarking of 79 models for T_g_ prediction, which revealed synergistic interactions between molecular fingerprints and neural architectures. Their study evaluated how different feature engineering strategies impact model performance, particularly highlighting the efficacy of Simplified Molecular Input Line Entry System (SMILES) derived molecular fingerprints (e.g., Morgan fingerprints with radius = 3 and nBits = 2048) when combined with neural architectures. He et al. [17] demonstrated the scalability of this approach by developing a quantitative structure–property relationship (QSPR) model for 695 polyesters, achieving experimental validation errors within 17.4 °C through virtual screening. Bo et al. [18] conducted a comprehensive study on polyimide (PI) materials, focusing on 11 key properties across four categories. They developed a high-throughput predictive framework incorporating diverse feature representations (e.g., Morgan fingerprints, Rational Design Kit (RDKit)/Mordred descriptors) and machine learning models. To elucidate the physicochemical mechanisms underlying model predictions, they applied SHapley Additive exPlanations (SHAP) analysis. By leveraging SHAP values to quantify feature importance, they identified critical structural determinants influencing each property at the molecular level. Building on these insights, they designed three PI variants with distinct structural features, demonstrating the practical utility of using SHAP for interpretability in guiding rational materials design. While these studies highlight the transformative potential of machine learning in polymer design, their success critically depends on large-scale, high-quality datasets [19,20,21,22]. In contrast, the BMI resin field is constrained by data scarcity [23,24,25] and label noise arising from inconsistent experimental conditions, severely limiting model predictive capabilities.

To address the data scarcity challenge in BMI resin research, we employ transfer learning, a paradigm where knowledge gained from large datasets is repurposed for related tasks with limited data [26]. This approach has demonstrated efficacy in materials informatics. Yamada et al. [27] achieved high predictive performance in material property estimation using only tens of samples through their XenonPy.MDL pre-trained model library. Zhang et al. [28] developed a transfer learning framework to predict the stress–strain curves of polymer composites, achieving a 46.14% accuracy improvement in plastic deformation stages through optimal transport integration. Kazemi-Khasragh et al. [29] extended this concept to diverse polymer property prediction, accurately forecasting thermal and mechanical properties using datasets as small as 13 samples. Building on these foundations, we propose a hybrid framework that combines transfer learning and interpretable machine learning to overcome data limitations in BMI resin studies. The framework leverages knowledge from a large-scale polymer database to compensate for data limitations in BMI systems while incorporating explainability techniques to unravel the structural determinants of T_g_. This approach aims to achieve the two following objectives: (1) enhancing predictive accuracy in data-scarce scenarios through transfer learning and (2) establishing a quantitative structure–property relationship for a rational molecular design of high-performance BMI resins.

## 2. Materials and Methods

### 2.1. Data Collection

A two-tier dataset architecture was employed. The base dataset (Data_1) comprised 3916 diverse polymers [30], among which 697 PI [31] were added due to their topological similarity with the imine pentacyclic structure of BMI to improve the pre-training effect of BMI-specific feature extraction. The target dataset (Data_2), constructed through experimental synthesis and literature curation [4,32,33,34,35,36,37,38,39,40,41,42,43,44,45,46,47,48,49,50,51,52,53], contained 78 BMI molecules, which is a scarcity that posed the primary challenge for model development.

**Figure 1:**
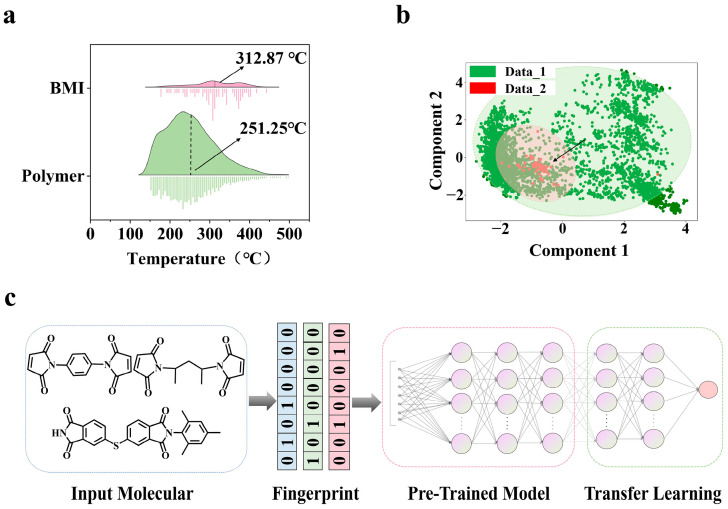
Data characterization and methodological framework. (**a**) Comparative kernel density estimation (KDE) profiles: Data_1 (green) shows multimodal distribution patterns, whereas Data_2 (red) exhibits elevated mean values attributed to rigid structural motifs; (**b**) principal component analysis (PCA) dimensionality reduction: green/red regions denote Data_1/Data_2 distributions; arrows indicate transfer learning pathways; (**c**) technical roadmap: molecular structure → Rational Design Kit (RDKit) fingerprint generation → deep neural modeling → transfer learning adaptation, forming a framework for cross-domain knowledge migration.

In order to systematically characterize the two-tier dataset architecture, we visualized the T_g_ distribution of the dataset (Figure 1a) using kernel density estimation (KDE) and distributional analysis. Here, the horizontal coordinates represent the T_g_ values corresponding to each data point, while the vertical coordinates indicate the frequency density. The green curve denotes Data_1, and the red curve denotes Data_2. Figure 1a reveals the multimodal distribution of Data_1 (the average value μ = 251.25 °C), reflecting its composition of diverse polymer families, as well as the right-skewed distribution of Data_2 (μ = 312.87 °C), suggesting rigid structural patterns specific to BMI resins. The distinct distributional differences between the two datasets underscore the uniqueness of BMI resins compared to other polymers, explaining why generalized polymer models cannot directly predict the T_g_ of BMI.

Molecular structures from both datasets were encoded as Morgan fingerprints (see Section 2.2 for details) and subjected to principal component analysis (PCA). This method projects high-dimensional feature relationships onto interpretable 2D scatter plots, where the horizontal and vertical axes represent the first two principal components after dimensionality reduction. As shown in Figure 1b, partial domain overlap exists between green Data_1 clusters and red Data_2 clusters, indicating transferable latent representations while preserving domain-specific characteristics. Critically, this structural overlap exists only in latent feature space, and all BMI molecules in Data_2 possess unique molecular scaffolds absent from Data_1. The black arrows symbolize the knowledge transfer pathway from the general polymer space to BMI-specific regions.

While curing conditions (e.g., temperature, post-cure duration) and environmental exposure factors (e.g., humidity, UV irradiation) are widely recognized as critical determinants of T_g_ in polymer systems, these variables were not systematically incorporated into our modeling framework due to inherent limitations in the literature data reporting consistency. Furthermore, while cross-linking frequency as a network structural parameter that directly affects T_g_ by restricting polymer chain movement, it was excluded from our analysis due to two practical limitations, namely (1) the experimental difficulty in measuring this property consistently across large datasets and (2) a lack of standardized measurement protocols between different studies. Although elevated cross-linking frequency is empirically linked to increased T_g_ in BMI systems, our study prioritized molecular-level descriptors to balance model complexity with data availability constraints. Additionally, we acknowledge that heterogeneity in T_g_ measurement methodologies across studies introduces unavoidable variability into the dataset. Such inherent noise contributes to model robustness by exposing the predictive framework to diverse measurement conventions, thereby enhancing generalization capabilities. This data collection approach reflects a deliberate trade-off between mechanistic completeness and pragmatic model applicability within the constraints of accessible experimental data.

### 2.2. Feature Engineering

A Morgan fingerprint [54] is a circular fingerprint encoding molecular substructures through hashed bit patterns. This encoding strategy preserves topological information at multiple scales while maintaining computational efficiency for neural processing. Unlike scalar molecular descriptors that aggregate global properties (e.g., molecular weight, the number of aromatic rings), Morgan fingerprints retain spatial relationships between functional groups, enabling neural networks to learn representations of structural features for T_g_ determination. The data preprocessing workflow (Figure 1c) utilized RDKit (RDKit: Open-source cheminformatics; http://www.rdkit.org (accessed on 11 March 2025)) to generate 2048-dimensional Morgan fingerprints from canonical SMILES [55] using the GetMorganFingerprintAsBitVect function with radius = 3 and nBits = 2048, capturing local chemical environments up to three bonds away while maintaining computational efficiency for neural processing.

### 2.3. Training Strategy of Transfer Learning Model

To address the sample scarcity in Data_2, a two-stage transfer learning framework was devised (Figure 1c). Stage 1 involved pre-training a multilayer perceptron (MLP) model on Data_1, featuring a 2048-dimensional input layer followed by three fully connected layers (1024/512/256 neurons with ReLU activation) and 30% dropout regularization. The model underwent 200 training epochs using the Adam optimizer with dynamic learning rate adjustment via ReduceLROnPlateau. Stage 2 implemented selective fine-tuning during transfer to Data_2: all parameters except the final five layers were frozen, enabling gradient updates only in the last two fully connected layers. This hierarchical adaptation mechanism preserved cross-domain generalizable features while enabling localized parameter tuning in Stage 2, where all parameters except the final five layers were frozen to allow gradient updates only in the last two fully connected layers. Hyperparameter optimization employed grid search with early stopping, exploring learning rates (1 × 10^−3^ to 1 × 10^−5^), batch sizes (16/32/64 samples/batch), and L2 regularization strengths (λ = 0.001). The final convolutional neural network (CNN) architecture comprised three convolutional blocks with 32/64/128 filters (3 × 3 kernels), augmented by a 0.3 dropout rate and batch normalization, trained using the Adam optimizer (1 × 10^−4^ initial learning rate). For the MLP, a three-layer fully connected network (1024/512/256 neurons) was configured with 0.3 dropout and L2 regularization, optimized via AdamW (5 × 10^−5^ learning rate). The deep neural network (DNN) adopted a five-layer sequential structure (1024/256/128/64/32 neurons) with 0.3 dropout, utilizing Adam optimization (1 × 10^−5^ learning rate). All experiments were executed on a Linux workstation with an NVIDIA RTX 4090 GPU using python 3.11. The presentation and calculation of the evaluation metrics are described in Supplementary Section S1. Evaluation metrics included the root mean squared error (RMSE), mean absolute error (MAE), mean squared error (MSE), and coefficient of determination (R^2^).

### 2.4. Virtual Structure Proposed

**Figure 2:**
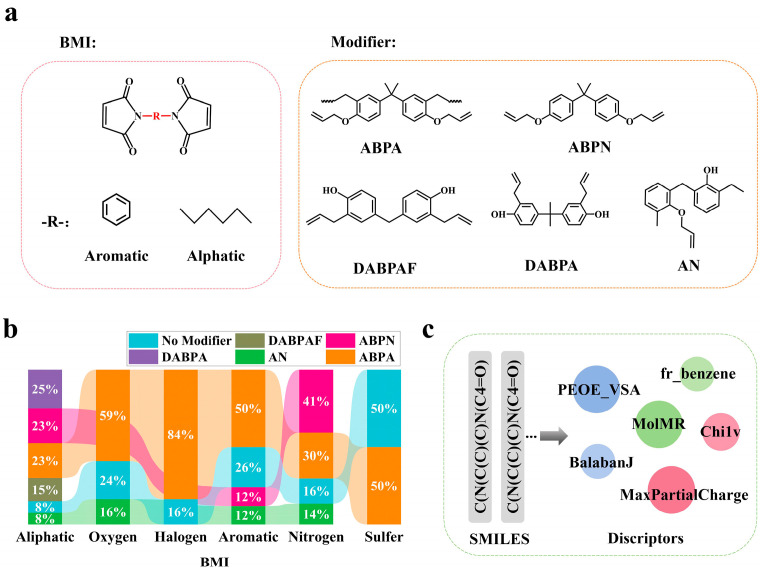
Bismaleimide-based polyimide (BMI) molecular design. (**a**) Classification framework based on R-group features (aromatic/aliphatic) and systematic functional group modification strategies; (**b**) modifier types showing functional group compositions in five copolymer structures and self-polymerized samples; (**c**) process of calculating descriptors.

In order to elucidate the structure–property relationship between BMI molecules and their T_g_, we categorized BMI molecules as aromatic (conjugated systems such as benzene rings) and aliphatic (linear or branched alkanes) based on the characterization of the R-groups, as shown in Figure 2a. We employed a multiscale functional group modification strategy by introducing thioether (-SH), nitrogen-containing groups (-NH_2_/NO_2_), oxygen-containing groups (hydroxyl, carbonyl, ester), and halogen substituents (F, Cl, Br, I). Additionally, five representative copolymer-modified architectures were incorporated, including ABPN, ABPA, DABPA, DABPAF, AN, and unmodified self-polymerization samples, to simulate real-world modification processes [56,57,58]. The functional group type distribution is visualized in Figure 2b, comprehensively reflecting the structural diversity of BMI resin macromolecules. The T_g_ prediction of these designed virtual structures using Model_1 yielded a virtual database of BMI (Data_3, n = 1092).

### 2.5. Descriptor Calculated

Unlike Morgan fingerprints, molecular descriptors are more suitable for analyzing and quantifying global physicochemical properties, so we use molecular descriptors as feature inputs in the interpretable modeling process instead of Morgan fingerprints. We used RDkit to calculate molecular descriptors (Figure 2c), including 67 descriptors such as the maximum partial charge value carried by the atoms in the molecule (MaxpartialCharge), Balaban’s topological index (BalabanJ), the sum of atomic molar refractivities (MolMR), and so on. The specific descriptors and their meanings are shown in Supplementary Section S2 (Table S1). A hybrid feature selection method combining the Pearson correlation coefficient identified 23 core descriptors for quantitative structure–property relationship modeling. The results of screening the characterization correlations using the Pearson correlation coefficient method are shown in Supplementary Section S3 (Figure S1).

### 2.6. Interpretable Model

We tried six machine learning algorithms. Random forest (RF) [59] operates as an ensemble method combining multiple decision trees via bagging and feature randomness, thereby reducing variance and improving generalization. Ridge regression [60] extends ordinary least squares by introducing L2 regularization to penalize large coefficients, effectively mitigating multicollinearity and overfitting. K-nearest neighbors (KNN) [61] follows a non-parametric, instance-based learning paradigm where predictions are derived from the weighted average of the target variable in the nearest training examples within the feature space. Bayesian regression (NB) [62] incorporates a probabilistic framework by assuming a prior distribution over model parameters, with predictions formulated as posterior distributions via Bayes’ theorem. Support vector regression (SVR) [63] extends the principles of support vector machines to regression tasks by mapping input features into a high-dimensional kernel space through nonlinear transformations. Extreme gradient boosting (XGBoost) [64] implements a gradient boosting framework that sequentially trains decision trees to correct residual errors, employing regularization terms and shrinkage to enhance robustness against overfitting while maintaining computational efficiency through parallel tree construction. We used 5-fold cross-validation in our model training. Specific parameter settings for the model training process are given in Supplementary Section S4.

### 2.7. SHAP Analysis

SHapley Additive exPlanations (SHAP) [65], a game theoretic framework rooted in cooperative game theory, was employed to decompose model predictions into feature contributions by quantifying Shapley values—the marginal impact of each molecular descriptor on T_g_ predictions. By aggregating local explanations across the dataset, SHAP generated globally interpretable insights through summary plots and force diagrams, enabling the visualization of both linear and nonlinear descriptor relationships. The final model leverages SHAP-derived descriptor importance rankings to construct a transparent structure–property map, where each molecular descriptor’s contribution to thermal transition behavior is represented.

## 3. Results and Discussion

### 3.1. Performance Comparison of Different Neural Network Frameworks as Pre-Trained Models in Transfer Learning

Given that the success of transfer learning hinges critically on the pre-trained model possessing robust generalization capabilities, we systematically compared the performance of different neural network frameworks within the transfer learning framework. We selected three distinct neural network frameworks for pre-trained model comparison, namely MLP, a CNN, and a DNN. MLP, as a fundamental feedforward neural network, excels in capturing nonlinear relationships within data through its fully connected layers, making it particularly suitable for processing high-dimensional sparse molecular fingerprint data, such as Morgan fingerprints. In this study, the MLP model, employing a three-layer hidden structure, demonstrated exceptional predictive capability on the test dataset of Data_2, achieving an R^2^ value of 0.59. In contrast, the CNN, renowned for its convolutional and pooling layers, excels in tasks like image recognition. However, within the transfer learning framework of this study, the CNN architecture did not surpass MLP in terms of predictive accuracy and generalization performance. This discrepancy might stem from the CNN’s proficiency in handling local features and spatial hierarchies, which may not be fully leveraged when dealing with high-dimensional sparse molecular data. The DNN, or deep neural network, enhances model representational power by increasing network depth. The DNN model adopted in this study comprised five hidden layers, enabling it to learn more complex feature representations. Despite its theoretical strong fitting capability, the DNN’s performance on the specific tasks and datasets of this study still slightly lagged behind MLP. This could be attributed to potential overfitting issues during DNN training, as well as challenges posed by data scarcity and quality heterogeneity in this study.

**Figure 3:**
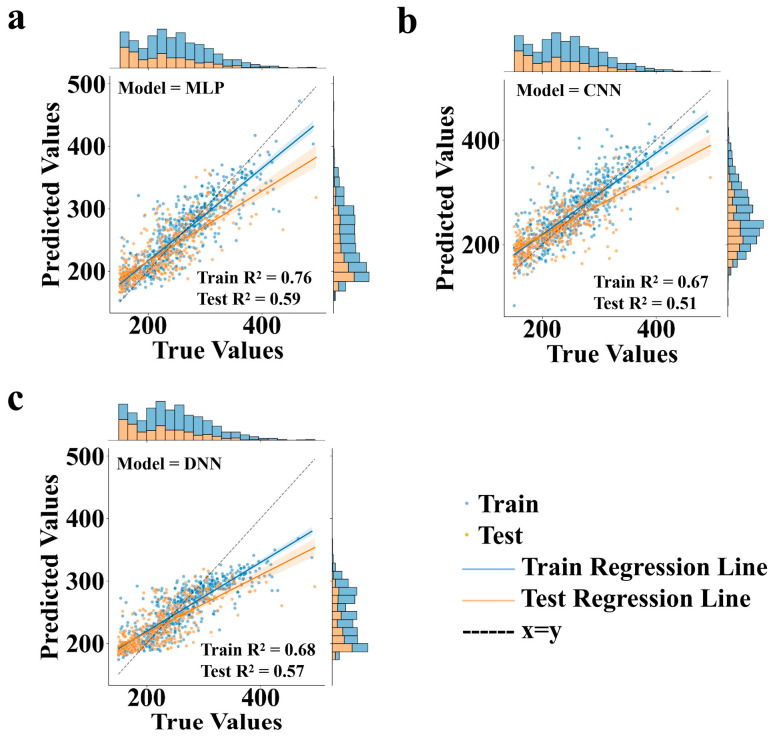
Pre-training performance comparison of neural network frameworks on Data_1: (**a**) multilayer perceptron (MLP) parity plot (training R^2^ = 0.76, test R^2^ = 0.59); (**b**) convolutional neural network (CNN) parity plot (training R^2^ = 0.67, test R^2^ = 0.51); (**c**) deep neural network (DNN) parity plot (training R^2^ = 0.68, test R^2^ = 0.57). Diagonal line indicates ideal prediction (x = y), with training/test data points and regression lines shown in blue/yellow.

To visually illustrate the performance disparities among these models, Figure 3 presents the parity plots for pre-training on the Data_1 dataset across all three models. In these plots, the horizontal axis represents the true values, while the vertical axis denotes the model predictions. The black dashed line signifies the x = y diagonal, where points closer to this line indicate predictions closer to the true values. The blue line represents the training regression line, and the yellow line denotes the test regression line. Blue dots correspond to training dataset points, and yellow dots to test dataset points. Figure 3a specifically depicts the parity plot for the MLP model trained on Data_1, with training and test R^2^ values of 0.76 and 0.59, respectively. Figure 3b,c showcases the parity plots for the CNN and DNN models, with training R^2^ values of 0.67 and 0.68 and test R^2^ values of 0.51 and 0.57, respectively.

The effectiveness of transfer learning hinges on the pre-trained model’s performance, particularly its generalization ability, as this directly impacts the subsequent fine-tuning process on Data_2. The superior performance of the MLP model in pre-training, as evidenced by its higher test R^2^ value and closer alignment of test data points to the diagonal line in Figure 3a, underscores its advantage in handling high-dimensional sparse molecular data. Based on this finding, we selected the MLP model as the pre-trained model, and subsequent transfer learning tasks and methodological explorations were all conducted based on the MLP model.

### 3.2. Necessity and Technical Advantages of Transfer Learning

Confronting the dual challenges of data scarcity (Data_2, n = 78) and quality heterogeneity in predicting the T_g_ of BMI, our proposed transfer learning framework (Figure 1c) demonstrates significant technical advantages. As our objective focuses on predicting the T_g_ of BMI resins, we allocated 10% of Data_2 as the test set (designated as Test_2) for comparative analysis across different modeling strategies. As our objective focuses on predicting the Tg of BMI resins, we allocated 10% of Data_2 as the test set (designated as Test_2) for comparative analysis across different modeling strategies. Due to the limited size of Data_2 and considering that allocating 10% for external testing is highly sensitive to the partitioning method, a stratified random sampling approach was employed to ensure statistical representativeness while maintaining class balance in this low-resource scenario. Table 1 systematically compares the performance of three MLP-based modeling strategies evaluated on Test_2, building upon the conclusion in Section 3.1 that MLP constitutes the optimal neural architecture for this task. As established, all three strategies utilize the MLP framework but differ in training paradigms, with (1) standalone training on Data_1 (general molecular database), (2) standalone training on Data_2 (n = 78 BMI-specific dataset), and (3) the two-stage transfer learning paradigm combining Data_1 pre-training with Data_2 fine-tuning. Specifically, the transfer strategy freezes all layers except the final five during fine-tuning, which is an optimal knowledge transfer mechanism validated in Section 3.3. To ensure methodological consistency, both the transfer learning and standalone Data_1 pre-training strategies utilized identical MLP architectures and hyperparameter configurations during their respective pre-training phases.

However, despite the initial expectation that Data_1—a large-scale general polymer dataset—would provide robust predictive capability (as evidenced by its R^2^ = 0.59 on internal testing in Section 3.1), the model’s performance on BMI-specific Test_2 plummeted to R^2^ = −6.19. This dramatic degradation stems from fundamental domain differences: BMI resins exhibit unique thermal behavior mechanisms distinct from conventional polymers, rendering generic structural patterns in Data_1 poorly transferable. While standalone training on Data_2 (n = 78) might seem a logical alternative, the resulting R^2^ = −4.10 and RMSE = 82.15 °C reflect inherent limitations: (1) extreme data scarcity prevents learning meaningful representations and (2) manual aggregation from heterogeneous literature sources introduces uncontrolled experimental noise, forcing the model to memorize spurious correlations rather than genuine structure–property relationships.

Faced with these challenges—Data_1’s domain mismatch and Data_2’s poor quality—the transfer learning framework strategically leverages Data_1’s generalizable physical information as foundational knowledge while adapting to BMI-specific features through fine-tuning. As shown in Table 1, the transfer learning approach achieved significant improvements in all evaluation metrics. There, MSE measures squared differences between predictions and true values; the RMSE represents the absolute error magnitude aligned with the target variable scale; the MAE directly reflects the average prediction deviation magnitude. All three metrics follow the “lower the better” principle. The R^2^ evaluates the model’s explanatory power for data variance, with values closer to one indicating better performance, while negative values signify worse performance than the baseline mean prediction. When evaluating the three distinct modeling strategies on Test_2, the transfer learning approach demonstrates superiority across all performance metrics. Notably, transfer learning improves R^2^ from −6.19 to 0.44 when compared to training on Data_1 alone, directly demonstrating the framework’s ability to correct for domain shifts. The RMSE decreases by 72.40% (from 97.53 °C to 27.27 °C), which indicates a significant improvement in real-world applicability. The results for the Data_2 standalone training show a negative R^2^ (−4.10) and considerable RMSE (82.15 °C), indicating that the model is not predicting accurately. This behavior stems from the model learning spurious correlations rather than true structure–attribute relationships in the small Data_2 dataset. The transfer learning framework utilizes the structural knowledge in Data_1 to effectively mitigate this issue, as evidenced by the positive R^2^ (0.44) and RMSE (27.27 °C) on Test_2. The consistent performance gains across the RMSE, MSE, MAE, and R^2^ metrics collectively validate the framework’s capacity to mitigate data scarcity limitations in predicting the T_g_ of BMI resin.

### 3.3. Optimizing Transfer Learning Performance Through Layer-Wise Fine-Tuning in MLP Architectures

**Figure 4:**
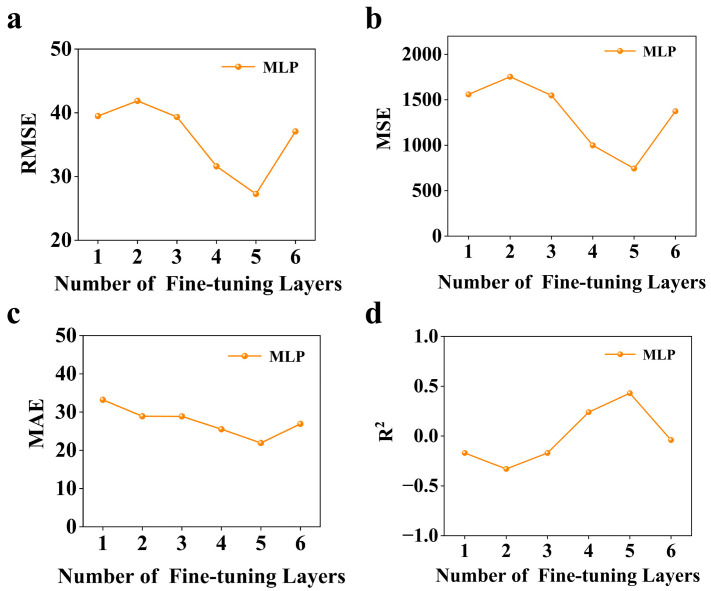
Layer-wise fine-tuning analysis for MLP-based transfer learning: (**a**) the root mean squared error (RMSE), (**b**) mean squared error (MSE), (**c**) mean absolute error (MAE), and (**d**) coefficient of determination (R^2^) performance trends across 1–6 fine-tuned layers. The MLP architecture comprises six layers (excluding input/output layers), with experiments systematically unfreezing 1–6 consecutive layers from the output end. The horizontal axis indicates the number of fine-tuned layers; vertical axes show error metrics (°C units for RMSE/MAE) and the coefficient of determination.

We explored the impact of varying fine-tuning layer counts on MLP-based transfer learning performance, with a particular focus on identifying the optimal balance between preserving pre-trained knowledge and adapting to target domain specifics. As shown in Figure 4, each subplot systematically evaluates a critical performance metric—the RMSE (4a), MSE (4b), MAE (4c), and R^2^ (4d)—along the vertical axis, while the horizontal axis spans the number of fine-tuned layers (ranging from 1 to 6). Given that the MLP architecture comprises six layers in total (excluding the input layer and output layer), our experiments systematically unfreeze one to six consecutive layers from the output end backward, enabling an investigation of adaptation effects.

The experimental curves reveal a consistent performance evolution pattern across all metrics. Initially, as layers are progressively unfrozen (moving from one to five layers), model performance improves markedly: the RMSE drops to 27.27 °C, the MSE decreases to 743.61, the MAE reduces to 21.92 °C, and R^2^ climbs to 0.44. This improvement phase peaks at five fine-tuned layers, indicating optimal adaptation where the model sufficiently adjusts higher-level representations for the target domain while retaining pre-trained feature extraction capabilities from the frozen initial layers. Beyond this optimal point, continued layer unfreezing (six layers) triggers performance deterioration across all metrics. This degradation suggests that excessive parameter adjustment may introduce domain-specific noise or disrupt previously learned robust features, negating the benefits of transfer learning.

These observations validate our strategy of adapting the last five layers. Such configuration creates a critical balance: maintaining frozen layers ensures stability in handling high-dimensional molecular data, while fine-tuning layers provides necessary flexibility for domain-specific calibration. The resulting pre-trained model, trained with this five-layer fine-tuning approach, represents the optimal intersection of transfer efficiency and adaptive capacity, achieving the highest predictive accuracy without compromising generalization capability.

### 3.4. Feature Interpretability Analysis

According to Section 3.3, the layer-wise fine-tuning strategy culminated in the development of Model_1, an optimized MLP architecture incorporating transfer learning principles that directly enabled the T_g_ predictions for BMI resins. To comprehensively elucidate the intrinsic physicochemical relationships governing the T_g_ of BMI resins, we conducted an interpretable machine learning analysis comparing experimental data (Data_2) and computationally augmented datasets (Data_3). While Morgan fingerprints excel in capturing structural patterns for predictive modeling, their inherent black-box nature limits physicochemical interpretability. Molecular descriptors, by contrast, encode quantifiable physicochemical properties, enabling direct correlation analysis between specific structural attributes and T_g_. This rationale motivated our selection of descriptors for interpretable model development.

Initially, we attempted to derive interpretable insights directly from Data_2, but the insufficient data quantity and methodological variability across experimental sources precluded reliable descriptor analysis. To overcome this limitation, we designed 1092 novel BMI structures, then employed Model_1 to predict their T_g_ values, thereby establishing the Data_3 dataset. Table 2 presents the quantitative performance metrics of six regression algorithms evaluated on both datasets. Notably, all models exhibited significantly improved predictive capabilities when trained on Data_3: for instance, the XGBoost model achieved a test set R^2^ of 0.63 with RMSE = 17.06 °C on Data_3 compared to R^2^ = −1.97 (RMSE = 48.35 °C) on Data_2. This dramatic performance disparity stems from Data_2’s limited sample size (n = 78) and inherent experimental heterogeneity across literature sources, which introduced confounding noise that compromised model generalization.

**Figure 5:**
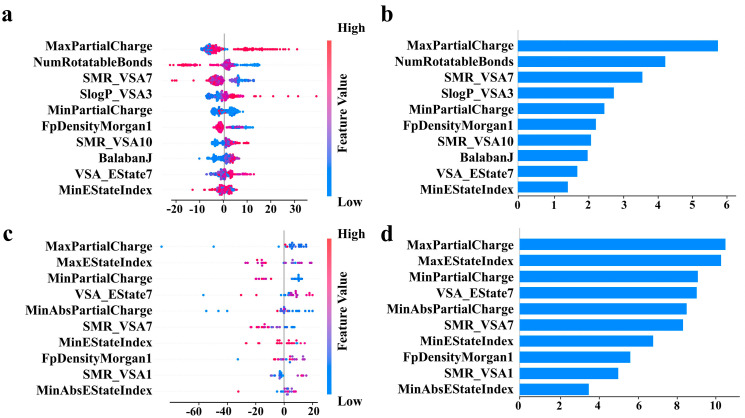
Feature role analysis: (**a**) SHapley Additive exPlanations (SHAP) summary plot for training interpretable models on Data_3; color shades indicate the degree of feature contribution, and red/blue represents the size of feature values; (**b**) feature importance ranking for training interpretable models on Data_3; the horizontal axis is the relative value of feature importance, and the vertical axis is the feature; (**c**) SHAP summary plot for training interpretable models on Data_2; color shades indicate the degree of feature contribution, and red/blue represents the size of feature values; (**d**) feature importance ranking for training interpretable models on Data_2; the horizontal axis is the relative value of feature importance, and the vertical axis is the feature.

According to the results in Table 2, given the superior performance of XGBoost on Data_3 (R^2^ = 0.63, RMSE = 17.06 °C), this model was selected for SHAP-based descriptor analysis. Figure 5 systematically shows the ordering of the effects and importance of different descriptors on the positive and negative correlations of T_g_. SHAP summary plots (Figure 5a,c) visualize feature contributions via color gradient encoding: red/blue tones indicate high/low feature values, with saturation intensity reflecting predictive impact magnitude. The feature importance ranking (Figure 5b,d) further quantifies the relative contribution of each descriptor, the horizontal coordinate represents the relative value of feature importance, and the vertical coordinate is each descriptor. For Data_3 (Figure 5a,b), the most important descriptors include MaxPartialCharge, which represents the maximum partial charge value carried by the atoms in the molecule. The fact that these descriptors rank as the most important indicates that the inhomogeneity of the charge distribution affects T_g_. MinPartialCharge represents the value of the smallest partial charge carried by an atom in a molecule. The importance ranking of MinPartialCharge is also high, and it works with MaxPartialCharge. A larger difference between the two represents a stronger localized concentration of charge within the molecule, which may lead to stronger electrostatic interactions between the molecules. It can be seen that MaxPartialCharge and MinPartialCharge have an important effect on the T_g_ of BMI; however, there is no obvious positive or negative correlation pattern. In addition, one of the most important descriptors is the number of rotatable bonds (NumRotatableBonds), which indicates the number of single bonds in the molecule that can be freely rotated. The molecules with fewer NumRotatableBonds have restricted chain segment mobility, more rigid molecular conformation, and higher T_g_. Meanwhile, the specific molecular surface area contribution (SMR_VSA7) and the specific surface area contribution to the lipid–water partition coefficient (SlogP_VSA3) also ranked high, indicating that the molecular surface area properties also affect T_g_, probably because they affect the stacking mode of the molecules and intermolecular interactions, which in turn have an effect on T_g_.

In order to verify the authenticity of the analysis results of virtual Data_3, we used Data_2 to train the XGBoost model for feature significance analysis and obtained results (Figure 5c,d) similar to those of Data_3. Notably, MaxPartialCharge, MinPartialCharge, representing charge inhomogeneity, and SMR_VSA7, representing molecular surface properties, are still in the top rank, but the model fails to capture the influences such as NumRotatableBonds, representing molecular topological complexity. This is directly related to the small number of datasets.

Two experimentally characterized BMI derivatives (BMI-I and BMI-II) sourced from the literature [66,67,68] were subjected to computational analysis (detailed in Supplementary Section S5, Tables S2 and S3). The Model_1 predictions yielded T_g_ values of 263.75 °C and 323.03 °C for BMI-I and BMI-II, respectively. It is important to note that the literature-reported experimental values for these derivatives are given as ranges (T_g_ > 260 °C and T_g_ > 300 °C) rather than specific numerical values. In this study, we have chosen to use the lower bounds of these ranges (260 °C and 300 °C) as reference points for comparison with our model predictions. Notably, the Model_1 predictions show excellent quantitative agreement with these conservative benchmarks, exhibiting absolute deviations of 3.75 °C (1.44%) and 23.03 °C (7.67%), respectively. The concomitant calculation of molecular descriptors revealed that while most topological parameters remained consistent between the two systems, the NumRotatableBonds metric exhibited a marked difference (22 and 16 bonds), inversely correlating with measured T_g_. This experimental and model comparison analysis not only validates Model_1’s predictive accuracy but also reinforces the structural complexity–T_g_ relationship posited by the interpretable framework, as reduced molecular flexibility (lower NumRotatableBonds) directly corresponds to evaluate T_g_, thereby substantiating molecular topology as a critical determinant influencing T_g_.

In summary, to rigorously validate our findings, we employed a two-step verification process. First, we established cross-dataset consistency through an independent analysis of experimental Data_2, confirming the dominant role of charge heterogeneity and molecular surface properties while highlighting limitations in capturing topological complexity. Second, we conducted targeted experimental validation using BMI-I and BMI-II, addressing the observed discrepancy and empirically substantiating the structural complexity–T_g_ relationship.

## 4. Conclusions

The hybrid framework proposed in this study integrates transfer learning and interpretable machine learning to successfully achieve the efficient prediction of Bismaleimide-based polyimide (BMI) glass transition temperature (T_g_). Through SHapley Additive exPlanations (SHAP) analysis, this study elucidates the core mechanism of molecular descriptors’ influence on T_g_. Specifically, topological complexity (represented by NumRotatableBonds), charge distribution properties (represented by MaxPartialCharge and MinAbsPartialCharge), and molecular surface properties (SMR_VSA7 and SlogP_VSA3) are identified as the dominant influencing T_g_ factors. This success can be attributed to the effective transfer of chemical spatial knowledge through transfer learning and the explicit resolution of higher-order interactions through SHAP analysis. Additionally, ten possible higher T_g_ structures are given based on the predictions of the model_1 (Supplementary Section S6, Table S4).

However, this study acknowledges certain limitations, particularly the exclusion of external variables such as processing parameters, which may affect the model’s generalizability under varying processing condition. Furthermore, while the current study primarily focuses on the influence of monomer structures on T_g_, we recognize the potential importance of network structural information, such as cross-linking frequency, in influencing T_g_. A higher cross-linking frequency generally leads to a denser network structure, thereby restricting the movement of polymer chains and resulting in an elevated T_g_. Future research could extend this work by incorporating processing parameters, molecular characteristics, and network structural information to comprehensively reveal the multi-scale regulation mechanisms of T_g_, further advancing the field of high-temperature-resistant polymer design with both predictive accuracy and mechanistic transparency. We believe that through these efforts, we can advance the development of T_g_ predictive models towards greater accuracy and practicality, thereby providing stronger support for the design and development of high-performance thermosetting polymers.

## Figures and Tables

**Table 1:** Performance comparison of MLP-based strategies on Test_2.

Metrics	Data_1 Standalone	Data_2 Standalone	Transfer Learning (from Data_1 to Data_2)
RMSE (°C)	97.53	82.15	27.27
MSE	9512.74	6747.92	743.61
MAE (°C)	89.49	79.47	21.92
R^2^	−6.19	−4.10	0.44

**Table 2:** Performance of six interpretable machine learning models on test dataset from Data_2 and Data_3.

Model	Dataset	RMSE (°C)	MSE	MAE (°C)	R^2^
RF	Data_2	43.18	1864.35	36.42	−0.81
	Data_3	17.32	299.97	12.27	0.62
Ridge	Data_2	37.39	1398.15	30.72	−0.36
	Data_3	21.75	472.98	15.33	0.40
KNN	Data_2	64.09	4107.56	48.96	−2.98
	Data_3	20.45	418.29	13.65	0.47
Bayesian	Data_2	37.39	1397.86	30.33	−0.36
	Data_3	21.60	466.51	15.18	0.41
SVR	Data_2	37.02	1370.61	30.39	−0.33
	Data_3	19.07	363.58	13.38	0.54
XGBoost	Data_2	48.35	2337.33	39.91	−1.97
	Data_3	17.06	290.98	12.09	0.63

## Data Availability

The data are available from the authors upon request.

## Supplementary Materials

The following supporting information can be downloaded at: https://www.mdpi.com/article/10.3390/polym17131833/s1, Table S1: Introduction to molecular descriptor names and related meanings. Figure S1: Feature correlation analysis after feature selection, the darker the blue color means the stronger the positive correlation, the lighter the blue color the negative number means the stronger the negative correlation, the number represents the correlation value. Table S2: Three experimental verifications of molecular structure in the literature. Table S3: The significant relevant descriptor values for the selected molecules. Table S4: Structures with higher predicted values of T_g_ in predicting the virtual structure of the design. The detailed datasets of these models can be found at https://doi.org/10.5281/zenodo.15481912 (accessed on 21 May 2025).
