# Supplementary material for: Transfer Learning-Enhanced Prediction of Glass Transition Temperature in Bismaleimide-Based Polyimides
Source: Polymers (Basel). 2025 Jun 30;17(13):1833. doi: 10.3390/polym17131833 (PMC12251919; doi:10.3390/polym17131833)
Supplement: Supplementary file 1 [file polymers-17-01833-s001.zip › polymers-3686938-supplementary.pdf]

Supporting Information

for

Transfer Learning-Enhanced Prediction of Glass Transition Temperature in Bismaleimide-Based Polyimides

Ziqi Wang,[1,2,3] Yu Liu,[1,2,3,*] Xintong Xu,[1,2,3,] Jiale Zhang,[1,2,3,] Zhen Li,[1,2,3] Lei Zheng,[1,2,3] and Peng Kang,[1,2,3,*]

[1]*School of Materials Science and Engineering, Beihang University, No. 37 Xueyuan Road, Beijing, 100191, China*

[2]*State Key Laboratory of Materials Intelligent Design, Beihang University, No. 37 Xueyuan Road, Beijing, 100191, China*

[3]*Tianmushan Laboratory, Yuhang District, Hangzhou 311115, China*

Corresponding authors:

E-mail: pengkang@buaa.edu.cn

E-mail: zhenglei@buaa.edu.cn

College of Materials Science & Engineering

Beihang University

Xueyuan Road. #37

Beijing,

100191, China

Contents

Section S1. Supplementary Materials for Evaluation Metrics..........................Page S3

Section S2. Supplementary Materials for Descriptors……....……………..……....Page S4

Section S3. Supplementary Materials for Feature Selection.............................Page S6

Section S4. Supplementary Materials for detailed parameter settings……………..……………….………………………......…...……..…….....Page S7

Section S5. Supplementary Materials for Molecular Structures in the literature...Page S8

Section S6. Supplementary Materials for Possible High T_g Structures…...Page S9

Section S1. Supplementary Materials for Evaluation Metrics

Mean Absolute Error (MAE) is a simple and intuitive metric that measures the average magnitude of the errors between predicted and actual values. MAE is not sensitive to extreme outliers because it uses absolute differences instead of squared differences.The formula for MAE is:

$$MAE = \frac{1}{n}\sum_{i=1}^{n}|y_i - \hat{y}_i| \quad \text{(S1)}$$

Where y_i is the actual value; $\hat{y}_i$ is the predicted value; n is the number of data points.

Root Mean Squared Error (RMSE) is a widely used metric for evaluating the accuracy of a predictive model. It measures the average magnitude of the errors between predicted values and actual values, giving more weight to larger errors due to the squaring operation. This makes RMSE particularly useful when it is important to penalize significant deviations from the true values.The formula for RMSE is:

$$RMSE = \sqrt{\frac{1}{n}\sum_{i=1}^{n}(y_i - \hat{y}_i)^2} \quad \text{(S2)}$$

Where y_i is the actual value. $\hat{y}_i$ is the predicted value.n is the number of data points.

Mean Squared Error (MSE) is similar to RMSE but does not take the square root. It is commonly used in optimization processes because it is differentiable, which facilitates the use of gradient-based algorithms for model training.The formula for MSE is:

$$MSE = \frac{1}{n}\sum_{i=1}^{n}(y_i - \hat{y}_i)^2 \quad \text{(S3)}$$

Where y_i is the actual value; $\hat{y}_i$ is the predicted value; n is the number of data points.

The Coefficient of Determination, denoted as R^2, is a statistical measure that represents the proportion of the variance in the dependent variable that is predictable from the independent variable(s). It provides an indication of how well the model fits the data, with values ranging from negative infinity to 1.The formula for R^2 is:

$$R^2 = 1 - \frac{\sum_{i=1}^{n}(y_i - \hat{y}_i)^2}{\sum_{i=1}^{n}(y_i - \bar{y})^2} \quad \text{(S4)}$$

Where y_i is the actual value; $\hat{y}_i$ is the predicted value; $\bar{y}$ is the mean of the actual values; n is the number of data points. .

Section S2. Supplementary Materials for Descriptors

Table S1. Introduction to Molecular Descriptor Names and Related Meanings

QED	Quantitative Estimate of Drug-likeness
MolWt	Molecular Weight: Sum of atomic weights of all atoms in the molecule (g/mol).
HeavyAtomMolWt	Heavy Atom Molecular Weight: Molecular weight excluding hydrogen atoms (g/mol).
ExactMolWt	Exact Molecular Weight: Molecular weight calculated using precise isotopic masses (not rounded).
NumValenceElectrons	Number of Valence Electrons: Total valence electrons in the molecule.
NumRadicalElectrons	Number of Radical Electrons: Total unpaired electrons in the molecule.
MaxPartialCharge	Maximum Partial Charge: Highest atomic partial charge (e.g., from Gasteiger/MMFF94 calculations).
MinPartialCharge	Minimum Partial Charge: Lowest atomic partial charge.
MaxAbsPartialCharge	Maximum Absolute Partial Charge: Largest absolute value of atomic partial charges.
MinAbsPartialCharge	Minimum Absolute Partial Charge: Smallest absolute value of atomic partial charges.
FpDensityMorgan1/2/3	Morgan Fingerprint Density: Fraction of bits set in Morgan circular fingerprints (radii 1, 2, 3).
BalabanJ	Balaban's Topological Index: A measure of molecular branching
BertzCT	Bertz Complexity Index: A topological index combining bond connectivity, atom types, and symmetry.
Chi0, Chi0n, Chi0v	Valence-Adjusted Connectivity Indices: Order-0 connectivity indices (variants normalized for size or valence).
Chi1, Chi1n, Chi1v	Valence-Adjusted Connectivity Indices: Order-1 connectivity indices.
Chi2n, Chi2v	Valence-Adjusted Connectivity Indices: Order-2 connectivity indices (normalized).
Chi3n, Chi3v	Valence-Adjusted Connectivity Indices: Order-3 connectivity indices (normalized).
Chi4n, Chi4v	Valence-Adjusted Connectivity Indices: Order-4 connectivity indices (normalized).
HallKierAlpha	Hall-Kier Electrotopological Index: A connectivity index incorporating atomic electronegativities.
Ipc	Information Content Index: A complexity metric based on Shannon entropy of the adjacency matrix.
Kappa1, Kappa2, Kappa3	Shape Indices: κ_1 (elongation), κ_2 (planarity), κ_3 (symmetry) derived from moments of inertia.
LabuteASA	Labute's Approximate Surface Area: Estimate of solvent-accessible surface area.
SMR_VSA	SMR-Weighted VSA: Van der Waals surface area weighted by molecular refractivity (e.g., SMR_VSA1, SMR_VSA3).

PEOE_VSA	PEOE-Weighted VSA: Van der Waals surface area weighted by partial charges (e.g., PEOE_VSA2, PEOE_VSA8).
VSA_EState	EState-Weighted VSA: Van der Waals surface area weighted by Electrotopological State indices.
TPSA	Topological Polar Surface Area: Sum of polar atom surface contributions .
MolLogP	Octanol-Water Partition Coefficient: Logarithm of the lipid-water partition coefficient (predicted).
MolMR	Molar Refractivity: Sum of atomic molar refractivities ($cm^3 \cdot g^{-1} \cdot 10^{-2}$).
HeavyAtomCount	Number of Heavy Atoms: Total non-hydrogen atoms in the molecule.
NOCount	Nitro Group Count: Number of nitro groups (NO_2).
RingCount	Total Ring Count: Number of rings in the molecular structure.
NumAliphaticHeterocycles	Aliphatic Heterocycle Count: Number of non-aromatic heterocyclic rings.
NumAliphaticRings	Aliphatic Ring Count: Number of non-aromatic rings (all-carbon).
NumHAcceptors	Hydrogen Bond Acceptors: Number of electronegative atoms (e.g., N, O) with lone pairs.
NumHeteroatoms	Heteroatom Count: Number of non-carbon atoms (e.g., N, O, S).
NumRotatableBonds	Rotatable Bond Count: Number of single bonds allowing free rotation (excluding terminal/ring bonds).
SlogP_VSA	SlogP-Weighted VSA: Van der Waals surface area weighted by atomic logP contributions (e.g., SlogP_VSA2).
fr_amide	Amide Group Count: Presence/count of amide functional groups ($RCONR_2$).
fr_C_O, fr_C_O_noCOO	Carbonyl Group Count: Total carbonyl groups (C=O) and carbonyl groups excluding carboxylates (COO^-).
fr_NH0	Non-Protonated Amine Count: Number of amine groups with no protonation (NH_0).
fr_imide	Imide Group Count: Number of imide functional groups ($RCONHCR^2$).
Max/MinEStateIndex	Max/Min Electrotopological State Index: Maximum/minimum values of atomic EState indices (encoding electronegativity, hybridization).
Max/MinAbsEStateIndex	Max/Min Absolute Electrotopological State Index: Maximum/minimum absolute values of EState indices.

.

Section S3. Supplementary Materials for Feature Selection

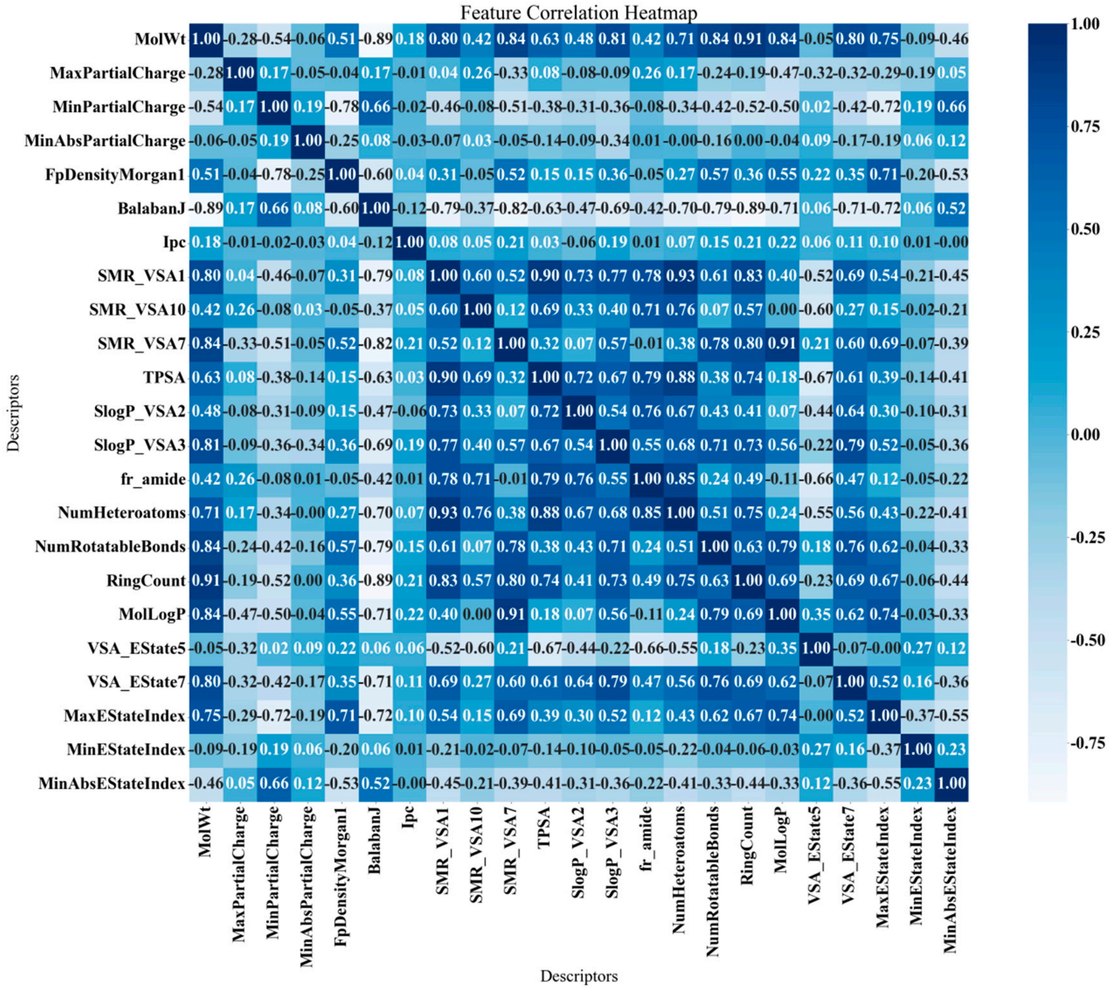

Figure S1. Feature correlation analysis after feature selection, the darker the blue color means the stronger the positive correlation, the lighter the blue color the negative number means the stronger the negative correlation, the number represents the correlation value.

Section S4. Supplementary Materials for detailed parameter settings

The parameter setting of training interpretable model on Data_2:
Parameter setting of RF:
Best Parameters: {'bootstrap': True, 'max_depth': 6, 'max_features': 'log2', 'min_samples_leaf': 2, 'min_samples_split': 2, 'n_estimators': 50}
Parameter setting of Ridge:
Best Parameters: {'alpha': 1000}
Parameter setting of SVR:
Best Parameters: {'C': 0.1, 'epsilon': 0.5, 'gamma': 'scale', 'kernel': 'poly'}
Parameter setting of KNN:
Best Parameters: {'n_neighbors': 3, 'p': 2, 'weights': 'uniform'}
Parameter setting of Bayesian:
Best parameters set found on development set:
{'priors': [0.5, 0.5], 'var_smoothing': 1.0}
Parameter setting of XGBoost:
Best Parameters: {'colsample_bytree': 0.8, 'learning_rate': 0.2, 'max_depth': 4, 'n_estimators': 50, 'reg_alpha': 0.1, 'reg_lambda': 1, 'subsample': 0.8}

The parameter setting of training interpretable model on Data_3:
Parameter setting of RF:
Best Parameters: {'bootstrap': True, 'max_depth': None, 'max_features': 'sqrt', 'min_samples_leaf': 1, 'min_samples_split': 5, 'n_estimators': 50}
Parameter setting of Ridge:
Best Parameters: {'alpha': 1}
Parameter setting of KNN:
Best Parameters: {'n_neighbors': 9, 'p': 2, 'weights': 'uniform'}
Parameter setting of Bayesian:
Best parameters set found on development set:
{'priors': [0.5, 0.5], 'var_smoothing': 1.0}
Parameter setting of SVR:
Best Parameters: {'C': 100, 'epsilon': 0.5, 'gamma': 'auto', 'kernel': 'rbf'}
Parameter setting of XGBoost:
Best Parameters: {'colsample_bytree': 0.8, 'learning_rate': 0.1, 'max_depth': 5, 'n_estimators': 100, 'reg_alpha': 0.1, 'reg_lambda': 10, 'subsample': 0.8}

Section S5. Supplementary Materials for Molecular Structures in the literature

Table S2. Three experimental verifications of molecular structure in the literature.

**Molecular**	**BMI**	**Modifier**	**Proportions**	**T_g(°C)**
I	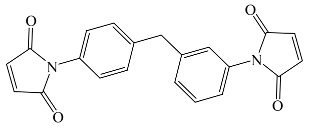	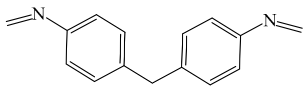	2:1	260
II	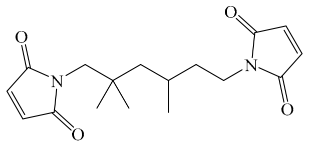	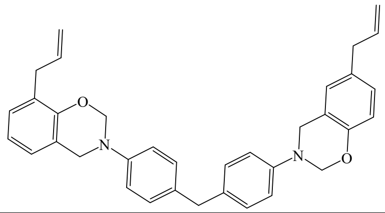	1:1	300

Table S3. The significant relevant descriptor values for the selected molecules.

Molecule	Predicted T_g (°C)	MaxPartialCharge	MinPartialCharge	SlogP_VSA3	NumRotatableBonds
BMI-I	263.75	0.339670787	-0.4800202	54.8580411	22
BMI-II	323.03	0.253112266	-0.472745917	45.10930479	16

Section S6. Supplementary Materials for Possible High T_g Structures

Table S4. Structures with higher predicted values of T_g in predicting the virtual structure of the design

SMILES	Temperature(°C)
CC(C1=CC=C(OCC=C)C=C1)(C)C2=CC=C(O/C=C/CC3C(N(C4=CC(O)=C(N(C5=O)C(C=C5)=O)C=C4O)C(C3)=O)=O)C=C2	380.8739736
CC(C1=CC=C(OCC=C)C=C1)(C)C2=CC=C(O/C=C/CC3C(N(C4=CC=C(C5=CC=C(N(C6=O)C(C=C6)=O)C=C5)C=C4N)C(C3)=O)=O)C=C2	425.7572808
CC(C1=CC=C(O/C=C/CC2C(N(CCN(C3=O)C(C=C3)=O)C(C2)=O)=O)C=C1)(C)C4=CC=C(O/C=C/CC5C(N(CCN(C6=O)C(C=C6)=O)C(C5)=O)=O)C=C4	399.6214867
CC(C1=CC=C(O/C=C/CC2C(N(C(C)(C)C(C)C(N(C3=O)C(C=C3)=O)(C)C)C(C2)=O)=O)C=C1)(C)C4=CC=C(O/C=C/CC5C(N(C(C)(C)C(C)C(N(C6=O)C(C=C6)=O)(C)C)C(C5)=O)=O)C=C4	392.312153
CC(C1=CC=C(OCC=C)C=C1)(C)C2=CC=C(O/C=C/CC3C(N(C4=CC=C(N(C5=O)C(CC5C/C=C/OC6=CC=C(C(C)(C)C7=CC=C(OCC=C)C=C7)C=C6)=O)C(Br)=C4Br)C(C3)=O)=O)C=C2	391.1658703
O=C(C=C1)N(C(C=C(C(N(C2=O)C(C=C2)=O)=C3)O)=C3O)C1=O	380.8739736
O=C(CC1C/C=C/OC2=CC=C(C(C)(C)C3=CC(CC)=C(OCC=C)C=C3)C=C2CC)N(C4=CC(Br)=C(N(C5=O)C(C=C5)=O)C=C4Br)C1=O	416.7328677
CC(C1=CC(CC)=C(OCC=C)C=C1)(C)C2=CC=C(O/C=C/CC3C(N(C4=CC=C(N(C5=O)C(CC5C/C=C/OC(C=C6)=C(CC)C=C6C(C)(C)C7=CC=C(OCC=C)C(CC)=C7)=O)C=C4O)C(C3)=O)=O)C(CC)=C2	395.9336854
CC(C1=CC(CC)=C(OCC=C)C=C1)(C)C2=CC=C(O/C=C/CC3C(N(C4=CC(O)=C(N(C5=O)C(CC5C/C=C/OC(C=C6)=C(CC)C=C6C(C)(C)C7=CC=C(OCC=C)C(CC)=C7)=O)C(O)=C4)C(C3)=O)=O)C(CC)=C2	383.4736716
CC(C1=CC(CC)=C(OCC=C)C=C1)(C)C2=CC=C(O/C=C/CC3C(N(C4=CC=C(N(C5=O)C(CC5C/C=C/OC(C=C6)=C(CC)C=C6C(C)(C)C7=CC=C(OCC=C)C(CC)=C7)=O)C=C4CCO)C(C3)=O)=O)C(CC)=C2	384.1004037
